# Research on Takeover Safety of Intelligent Vehicles Based on Accident Scenarios in Real-Vehicle Testing

**DOI:** 10.3390/s25175589

**Published:** 2025-09-07

**Authors:** Pingfei Li, Meiling Zhou, Chang Xu, He Li, Wenhao Hu, Zhengping Tan, Lingyun Xiao, Xiaojun Mou, Hao Feng

**Affiliations:** 1 School of Automation Engineering, University of Electronic Science and Technology of China, Chengdu 611731, China; xhlpf12@mail.xhu.edu.cn; 2 School of Automobile and Transportation, Xihua University, Chengdu 610039, China; 13258113540@163.com (M.Z.); xuchg99@163.com (C.X.); 15283767795@163.com (H.L.); tanzp@mail.xhu.edu.cn (Z.T.); qcshdj@gmail.com (X.M.); 3 Vehicle Measurement, Control and Safety Key Laboratory of Sichuan Province, Xihua University, Chengdu 610039, China; 4 Chongqing Changan Automobile Co., Ltd., Chongqing 400023, China; 5 SAMR Defective Product Recall Technical Center, Beijing 100101, China; xiaoly@samrdprc.org.cn; 6 Academy of Forensic Science, Shanghai 200063, China

**Keywords:** intelligent vehicle safety, human–machine cooperative driving, hazardous scenarios, takeover request, test verification

## Abstract

**Highlights:**

**What are the main findings?**

- Identified critical safety risks in intelligent vehicles during large-curvature curves and static obstacle scenarios, including lane departure and missed target detection;
- Quantified minimum driver reaction times for safe takeover: 4.12 s for longitudinal control and 1.87 s for lateral control.

**What is the implication of the main finding?**

- Emphasizes enhancing driver engagement during human-machine cooperative driving (HMCD) phases to reduce passive fatigue and improve emergency readiness;
- Proposes strengthening early warning systems in autonomous driving to ensure sufficient takeover time, addressing functional limitations in current driving automation systems.

**Abstract:**

With the increasing emergence of intelligent vehicles, novel accident patterns have gradually emerged. In human–machine cooperative driving (HMCD) states, despite driving automation systems being capable of controlling lateral and longitudinal vehicle motions over extended periods, functional limitations persist in specific scenarios due to insufficient expected functionalities. When combined with risk factors, such as driver distraction, these limitations significantly elevate accident risks. This study investigated takeover safety through real vehicle testing in two typical accident scenarios: large-curvature curves and static obstacles. The key findings include the following: (1) in scenarios involving large curvature curves and static obstacles, vehicles are prone to lane departure and missed target detection, which are typical dangerous scenarios; (2) during the human–machine cooperative driving phase, the design of the driving automation system should focus on enhancing driver engagement in driving tasks, while in the autonomous driving phase, the vehicle’s early warning capabilities should be strengthened; (3) the takeover request for longitudinal control requires at least 4.12 s of driver reaction time, while the takeover request for lateral control requires at least 1.87 s. This study provides important theoretical and practical references for safety in designing assisted driving systems and the testing of hazardous scenarios.

## 1. Introduction

With the rapid development of intelligent connected vehicle (ICV) technology, driving automation is currently transitioning from SAE Level 2 (partial automation) to Level 3 (conditional automation). During this transition phase, human–machine co-driving (in scenarios where fully autonomous driving cannot be guaranteed, the task of driving the vehicle is shared by the natural driver and the autonomous driving system) remains the dominant driving mode for intelligent vehicles [1]. According to statistics, the market penetration rate of ICVs equipped with combined assisted driving functions reached 47.3% in 2023 [2]. Notably, higher market penetration rates are associated with a significant reduction in traffic conflicts, which tend to decrease exponentially as penetration increases [3]. Despite the increasing adoption of automation technology, current systems still face considerable limitations in scenario recognition and human–machine interaction, resulting in emerging accident types and growing safety concerns. Investigations by the U.S. National Transportation Safety Board (NTSB) have revealed that existing driving automation systems often fail to adequately recognize or respond to specific scenarios. Combined with driver distraction caused by overreliance on automation, the handover process—when control must be returned to the driver after the system exceeds its operational limits—poses serious risks and has become a key contributing factor in traffic accidents [4].

In this context, accurate target recognition has become a fundamental prerequisite for understanding complex traffic scenarios, making sound decisions, and executing effective control. In challenging environments such as curved roads, slopes, or adverse weather, poor or failed target recognition can lead to misjudgment or delayed responses, directly increasing risk. Such failures can also extend the driver’s takeover reaction time, significantly raising the likelihood of accidents. Therefore, enhancing the accuracy and performance of target recognition is a critical strategy for mitigating accident risks. Concurrently, excessive driver trust in automation can also lead to safety hazards. Studies show that when automated systems are engaged, drivers often overestimate the system’s capability to handle complex or unexpected scenarios. This results in a passive monitoring state (‘passive vigilance’) and a diminished willingness to actively observe the road [5]. This mental complacency, combined with physical distractions (e.g., using mobile devices or engaging in conversations), compromises situational awareness and delays the driver’s ability to respond effectively when the system suddenly fails—such as during target-recognition errors—thus increasing accident risks.

Faced with the dual challenges of “perceptual failures triggering emergency takeovers” and “driver distraction delaying responses,” the scientifically defined takeover time (TOT)—the time interval between the system issuing a takeover request (TOR) and the driver regaining full control—has become a key factor in balancing system safety and usability [6]. Research shows that TOT directly impacts the system’s safety margin under high-risk scenarios (e.g., sufficient braking distance after delayed curve recognition) and the driver’s available time to regain perception and execute control, particularly in distracted states. For example, even when a takeover request is issued 5 s before a potential collision, distracted drivers still show decreased vehicle control stability and increased risk of longitudinal collisions [7]. To improve takeover safety, researchers have proposed pre-alert strategies that provide advance warnings in certain scenarios, such as highway exits. Evidence suggests that these strategies can significantly reduce driver braking reaction time, but they require a minimum takeover window of 7 s to be effective [8,9]. As a result, a dynamic optimization approach for the takeover threshold—based on target-recognition performance and driver cognitive load—has emerged as a vital technical strategy for addressing the limitations of current Level 2 autonomous systems.

Ultimately, the effectiveness of automated driving systems hinges on two key factors: the accuracy of environmental perception and the level of driver distraction. In urgent takeover scenarios, system safety must be evaluated with a holistic view of both system capabilities and human factors. To assess the system’s ability to accurately identify potential hazards and issue timely alerts, it is essential to conduct evaluations in realistic testing environments. In this regard, testing based on authentic accident scenarios has become a critical tool for system validation and improvement.

### 1.1. Related Work

Currently, numerous scholars have conducted research on the accuracy of detecting target objects, and the core competitiveness of both domestic and international companies primarily lies in the development of target detection algorithms. With advancements in computing power, algorithm design, and the refinement of datasets, research has gradually transitioned from traditional detection methods to those based on neural networks and deep learning technologies [10]. The rapid progress of deep learning has led to the emergence of two major technical approaches in object detection: two-stage detectors (e.g., Faster R-CNN) and one-stage detectors (e.g., YOLO and SSD) [11]. Although both approaches have yielded promising results, several challenges persist—particularly in detecting small objects, occluded targets, and handling multiple objects simultaneously [12]. Some studies, such as [13,14], have leveraged deep learning algorithms to improve detection accuracy but have not effectively addressed multi-class object detection. In the domain of pedestrian detection within crowded environments, the Double-Mask R-CNN method was proposed to reduce false negatives in [15]. Meanwhile, Ref. [16] focused on detecting objects in complex scenes, though this study did not assess detection accuracy. Existing research has primarily tested static and dynamic objects like pedestrians and vehicles, but there remains limited attention to small-scale objects, such as children and animals, where false negatives and false positives are more likely to occur.

To improve recognition accuracy for small objects, Ref. [17] enhanced detection algorithms, boosting both accuracy and robustness in complex and extreme environmental conditions. Addressing the issue of missed detections and false alarms, Ref. [18] proposed algorithmic improvements to reduce pedestrian detection failures in complex scenes, demonstrating reduced loss rates alongside improved accuracy. However, the effectiveness and robustness of these algorithmic optimizations must be validated under real-world driving conditions. Relying solely on simulation modeling fails to fully capture the dynamic variability and edge-case complexity of real road environments. Thus, real-world vehicle testing is a crucial and irreplaceable component for verifying the reliability of autonomous driving systems.

In this regard, scenario-specific real-vehicle testing becomes especially important—for example, in conditions involving curves and slopes. Ref. [19] compared 22 mainstream autonomous driving simulators and concluded that although simulation offers safe and efficient testing environments, the realism of real-vehicle testing remains irreplaceable. Researchers, including [20,21], conducted real-vehicle experiments on Level 2 driver-assistance systems in curved-road scenarios, emphasizing the importance of allocating adequate takeover time for drivers and improving lateral stability. Ref. [22] examined the impact of longitudinal highway slopes on driver performance through real-world testing.

From the aforementioned studies, it is evident that current research predominantly focuses on single scenarios with isolated target types. In summary, while progress has been made in real-vehicle testing within specific scenarios such as curves and slopes, these efforts remain limited to single-object, single-scenario tests. They fail to fully reflect the complexity and dynamic interactions of real-world road environments. In reality, multiple potential threats—such as pedestrians, vehicles, and unexpected obstacles—may appear simultaneously, or scene elements may change abruptly (e.g., a hidden obstacle emerging on a curve, or a blind spot appearing at the crest of a slope). Such complexity significantly increases system uncertainty and is likely to push the system beyond its operational boundaries, necessitating urgent driver takeovers. Therefore, ensuring safe and efficient driver takeovers in complex, multi-object, dynamically evolving scenarios has become a critical challenge that must be addressed to support the practical deployment of autonomous driving systems.

### 1.2. Problem Research

Drawing upon existing research in the field, it is evident that intelligent vehicle driving automation currently faces two fundamental safety challenges: (1) limited environmental perception and recognition capabilities in specific scenarios, and (2) risks arising from human misuse and the safety of system takeovers. These emerging types of accidents introduce new complexities for traditional accident investigation and risk assessment methodologies, underscoring the need for targeted research efforts.

This paper conducts a comprehensive investigation into typical accident patterns associated with automated driving, grounded in real-world traffic accident data. Through this analysis, two representative high-risk scenarios—high-curvature bends and static obstacles—are identified. To evaluate system performance under these conditions, controlled real-vehicle experiments are carried out in a closed testing environment. These experiments systematically assess the operational reliability of the automated driving system and provide an in-depth analysis of takeover safety dynamics. This study aims to offer theoretical support and practical guidance for improving the safety performance of intelligent vehicles. By integrating accident data analysis, scenario-based testing, and system performance evaluation, it examines driver takeover time in real-world traffic environments, thereby facilitating the development of more stable and responsive autonomous driving systems.

## 2. Materials and Methods

### 2.1. Research Methods

**Figure 1:**
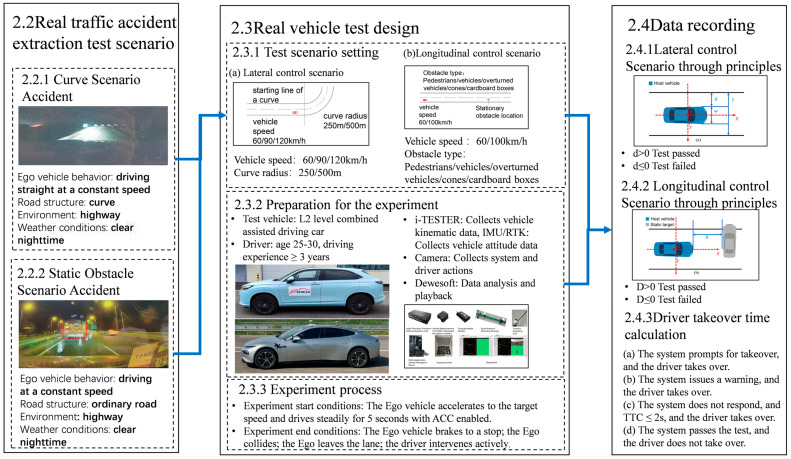
Technology roadmap.

To evaluate drivers’ takeover performance under real-world driving conditions involving potential hazards, this study first identified two representative test scenarios based on traffic accidents involving advanced driver-assistance systems (ADAS): a curve scenario representing lateral control challenges and a static-obstacle scenario representing longitudinal control challenges. Real-vehicle tests were then conducted using Level 2 combined assisted driving vehicles from two different manufacturers at a closed test facility. The primary focus was to assess the vehicles’ capability to safely navigate these scenarios and analyze drivers’ takeover behavior when the Adaptive Cruise Control (ACC) system was engaged. The specific research procedures are illustrated in Figure 1.

### 2.2. Typical Accident Scenarios

#### 2.2.1. Curved-Road Accident Scenario

**Figure 2:**
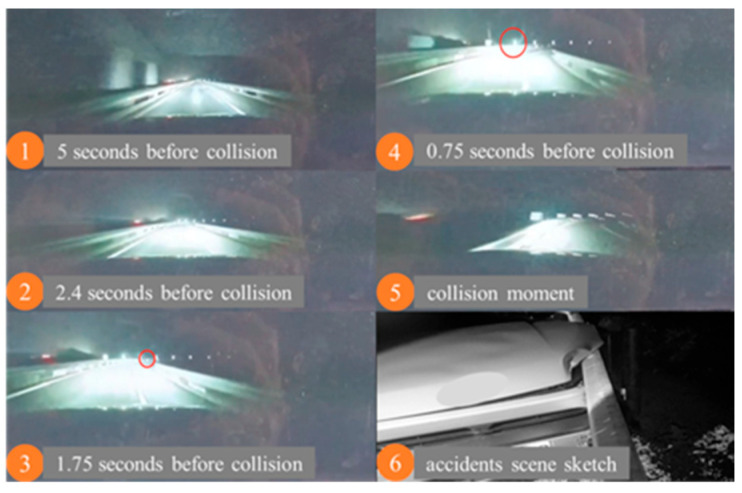
Schematic diagram of lateral control accident. (The red circle is the final location of the accident).

In October 2022, at 7 p.m., under clear-weather conditions with poor ambient lighting, an accident occurred on a curved section of a highway with no visual obstructions. A vehicle with Level 2 combined driving assistance activated (Navigate on Autopilot, NOA) was involved in an accident. As the driver’s destination required exiting the highway, the vehicle autonomously changed lanes from the main lane to the right curve ramp under the control of the assisted driving function. However, the vehicle did not decelerate, and the assisted driving state was deactivated without any warning or takeover prompt. The driver failed to take over the vehicle in time, only making an emergency evasive maneuver right before the collision, resulting in the vehicle colliding with the guardrail. The accident process is shown in Table 1 and Figure 2.

#### 2.2.2. Static-Obstacle Accident Scenario

**Figure 3:**
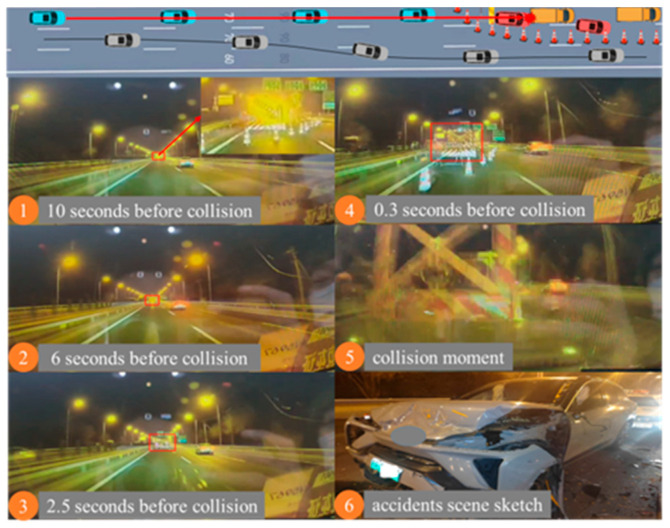
Schematic diagram of longitudinal control accident. (The red box is the final location of the accident).

In December 2022, at 11 p.m., under clear weather conditions with good ambient lighting from streetlights, an accident occurred on a straight section of an urban expressway with no visual obstructions. A vehicle with Level 2 combined driving assistance activated (NOA) was involved in an accident. Ahead of the vehicle, there was a temporary construction zone with static obstacles, including traffic cones, construction signs, engineering vehicles, and tail lights on the engineering vehicle. The vehicle had been traveling in the leftmost lane and only took emergency braking and steering measures when approaching the construction zone, but failed to avoid a collision, resulting in a rear-end collision with the engineering vehicle. No warning or takeover prompt was issued before the collision. The accident process is shown in Table 2 and Figure 3.

The two accidents share two typical characteristics: first, the driver’s over-reliance on the driving automation system; second, the vehicle failed to issue any warning or takeover prompt before the collision.

### 2.3. Real-Vehicle Testing Design

To address the curved-road and static-obstacle scenarios observed in the accidents, this study constructed actual scenarios in a closed environment and conducted real-vehicle testing using vehicles with Level 2 combined driving-assistance functions to analyze the performance of driving automation functions in typical hazardous scenarios.

#### 2.3.1. Test Scenario Setting

**Figure 4:**
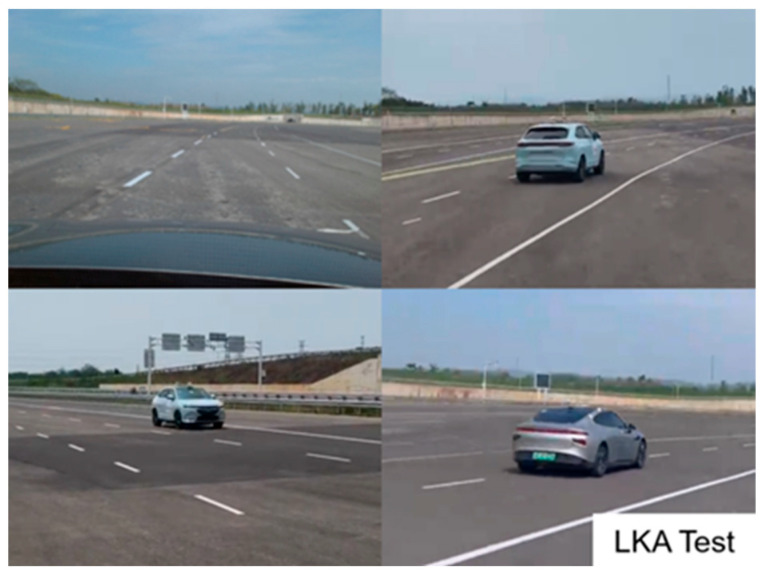
Lateral control test site.

**Figure 5:**
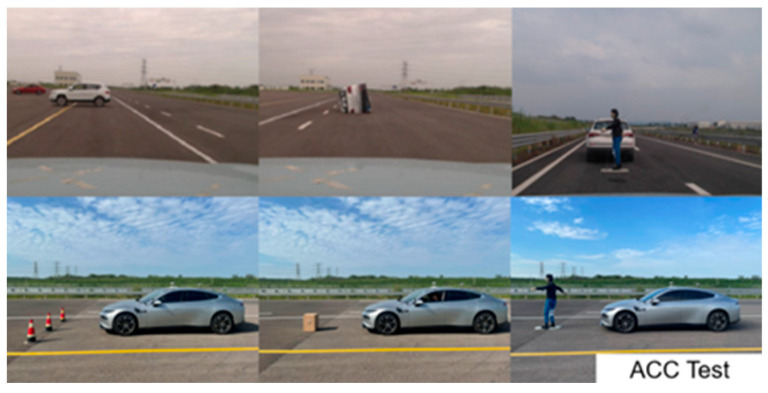
Longitudinal control test site.

Based on the accident analysis, two types of test scenarios were designed: a scenario involving a straight entry into a large curvature (lateral control capability test) and a scenario involving a straight road with a static obstacle ahead (longitudinal control capability test). The specific scenario parameters are shown in Table 3 and Table 4, and the test sites are shown in Figure 4 and Figure 5.

To fully test the vehicle’s lateral control capability, the main vehicle speed in the 500 m curve scenario was increased to 90 km/h and 110 km/h within the speed limit.

#### 2.3.2. Preparation for the Experiment

**Figure 6:**
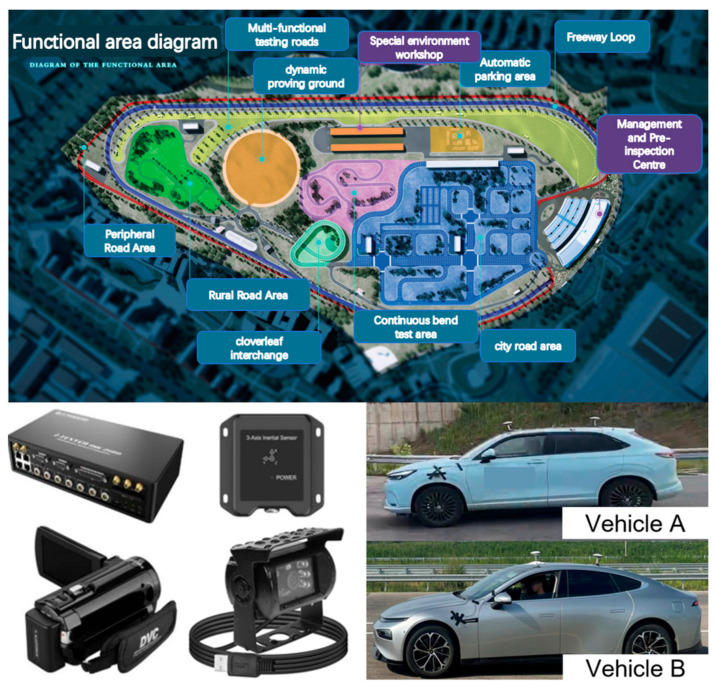
Test site and equipment.

The testing was carried out at the Chengdu Sino-German Intelligent Connected Vehicle Sichuan Test Base, utilizing both the multifunctional test track and high-speed loop. Data collection was conducted using the i-TESTER AVE 2100 high-precision acquisition system (China Automotive Engineering Research Institute, Chongqing, China), which comprises a high-precision positioning host, an IMU inertial navigation system, an RTK module, and Dewesoft 7.21 data acquisition and analysis software. The positioning host was connected to the vehicle’s CAN bus and IMU interface to record vehicle-side data, supporting data acquisition and storage at a frequency of 100 Hz. The IMU and RTK modules were responsible for capturing vehicle motion status and positional information, while the Dewesoft software facilitated data playback, analysis, and export. All the tested vehicles were Level 2 conditionally automated vehicles equipped with combined longitudinal and lateral assistance functions, as illustrated in Figure 6. Given the variation in advanced driver assistance systems (ADAS) function nomenclature across the two vehicle models, the terms “lateral control” and “longitudinal control” are used generically in this study to describe corresponding functionalities. Considering cost and testing precision, the sensor and function configurations of the tested vehicles are presented in Table 5. During testing, two young, experienced drivers were stationed inside the vehicles to supervise ADAS operation, having been fully briefed on the testing procedures in advance. Each scenario was repeated three times, and the final results represent the average values across these runs.

#### 2.3.3. Experiment Process

(a) Lateral Control Scenario

Before the experiment begins, the test vehicle accelerates from a standstill to the target speed and activates the ACC function. After driving steadily on a straight road for 5 s, it enters a curve. The experiment ends when the vehicle has driven along the curve for 5 s or exits the lane.

(b) Longitudinal Control Scenario

Before the experiment begins, the test vehicle accelerates from a standstill to the target speed and activates the ACC function. Data recording begins when the vehicle is 200 m away from the target object. The experiment ends when the vehicle decelerates and stops before the target object, when it collides with the target object, or when the TTC between the main vehicle and the target vehicle reaches 2 s, and the intelligent driving-assistance system fails to control the vehicle to brake, prompting the driver to actively swerve to avoid the collision.

### 2.4. Data Recording

#### 2.4.1. Lateral Control Scenario Through Principles

**Figure 7:**
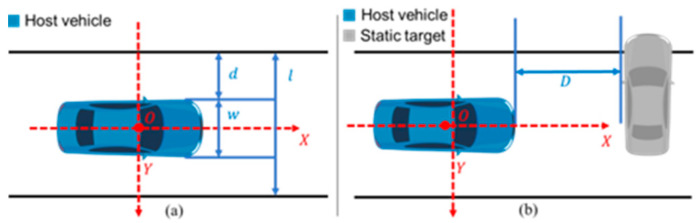
Schematic diagram of lateral and longitudinal distances. (**a**) lateral distances; (**b**) longitudinal distances.

As shown in Figure 7, a coordinate system was established with the inertial navigation device installed on the roof as the origin O, the vehicle centerline as the X-axis, and the lane line connection as the Y-axis. The lane width l was 3.6 m. Data collected after the activation of the vehicle’s lateral and longitudinal control functions were extracted, and the data were verified based on synchronized video information from the vehicle’s onboard cameras.

In the lateral control capability test, the lateral distance d between the left front wheel and the left lane line was collected:

(1) $$d=\frac{l-w}{2},$$

where l is the lane width (m), and w is the vehicle width (m).

Based on the video and data, the lateral distance $d_{f}$ at which the vehicle issued a lane-departure warning and the critical departure time $T_{f}$ (Time To Departure) were obtained. Since the test vehicles have different widths, and studies have shown that the acceptable lateral oscillation amplitude for future autonomous vehicles is 0.48 m [23], the ideal lateral distance $d_{p}$ considering the oscillation amplitude was calculated using Equation (1), as shown in Table 6.

#### 2.4.2. Longitudinal Control Scenario Through Principles

In the longitudinal control capability test, the longitudinal distance d from the front of the vehicle to the static obstacle was collected. Based on the collected data, the Time To Collision (TTC) was calculated:

(2) $$T=\frac{D}{V}$$

where T is the collision time (s); D is the longitudinal distance (m), and V is the vehicle speed (m/s).

Based on the time difference between the warning and takeover in each scenario, the takeover reaction time ∆T was calculated:

(3) $$∆T=T_{2}-T_{3},$$

where $T_{1}$ is the warning time (s); $T_{2}$ is the takeover request time (s), and $T_{3}$ is the takeover time (s).

#### 2.4.3. Driver Takeover Time Calculation

During the entire testing process, based on the driving state of the combined assisted driving system, the driver’s takeover timing followed the principles below:

(1) When the system issued a warning but did not actively respond, the driver took over vehicle control;
(2) When no warning was issued, but the ACC function disengaged, the driver took over vehicle control;
(3) When neither a warning was issued nor the ACC function disengaged, and the system failed to respond effectively to the target ahead, the driver took over control when the Time To Collision (TTC) was ≤2 s;
(4) If the system responded effectively to the obstacle and continued to drive within the lane, the driver did not take over, and the system maintained control.

## 3. Results

The test results show that neither of the two test vehicles triggered a takeover prompt during the entire testing process, but the driving-assistance system issued warnings in some test scenarios. The main reason for this phenomenon is that the test scenarios were designed based on real traffic accidents, which are highly dangerous. Therefore, in the subsequent analysis, the moment when the test vehicle issued a warning was considered equivalent to a takeover prompt; that is, the takeover reaction time can be equivalent to $∆T=T_{1}-T_{3}$, and the analysis was conducted based on this assumption.

### 3.1. Analysis of Lateral Control Capability Test Results

**Figure 8:**
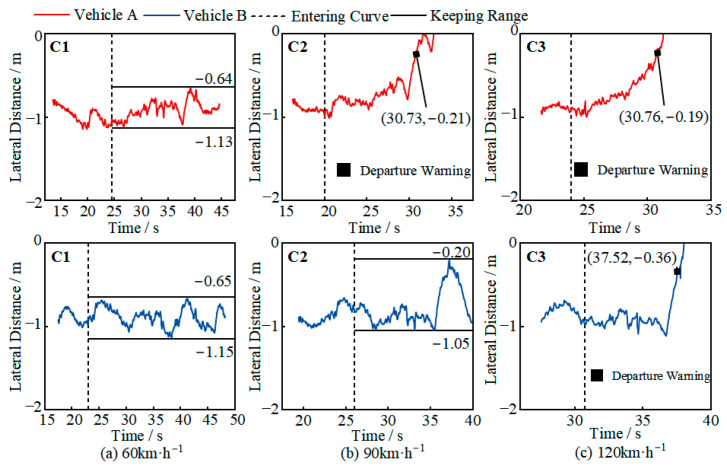
An analysis of the R250M curve test results (negative lateral distance indicates that the car is within the lane).

The test results for the R250M curve scenario are shown in Figure 8. At 60 km/h, both vehicles were able to maintain their position in the curve for more than 10 s, with lateral distances ranging from 0.64 to 1.13 m and 0.65 to 1.15 m, respectively, close to the ideal range. At 90 km/h, Vehicle A maintained its position for 7 s after entering the curve, but as it approached the constant curvature section, its lateral control system could not prevent lane departure, and the vehicle gradually drifted out of the lane. Vehicle B, after entering the constant curvature section, showed a decreasing lateral distance and a tendency to drift out of the lane, but its lateral control system was able to suppress the drift and keep the vehicle within the lane, with a lateral distance range of 0.20–1.05 m. However, the vehicle’s oscillation amplitude in the curve was large, which could affect the safety of the driver or other traffic participants. At 120 km/h, the lateral control systems of both Vehicle A and Vehicle B were unable to suppress the lateral movement after entering the curve, and the vehicles eventually drifted out of the lane. Since the testing was conducted in a closed environment with sufficient safety conditions, the driver did not take over the vehicle.

**Figure 9:**
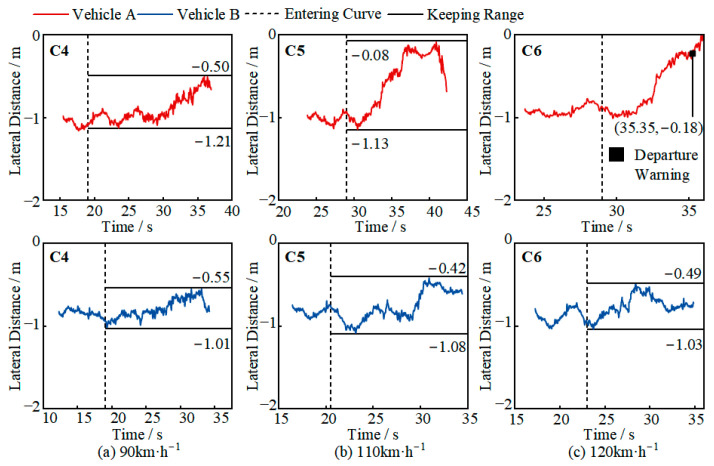
R500M curve test results (negative lateral distance indicates that the car is within the lane).

The test results for the R500M curve scenario are shown in Figure 9. At 90 km/h, both Vehicle A and Vehicle B were able to maintain their position in the curve for more than 10 s, with lateral distances ranging from 0.50 to 1.21 m and 0.55 to 1.01 m, respectively. At 110 km/h, Vehicle A barely managed to stay within the lane, drifting close to the left lane line before returning to the lane, with a lateral distance range of 0.08–1.13 m. Although the system did not issue a warning and was able to suppress the drift, the vehicle’s oscillation amplitude was too large. At 120 km/h, Vehicle A drifted out of the lane immediately after entering the curve and could not complete the test. In scenarios C5 and C6, Vehicle B was able to maintain its position in the curve for more than 10 s, with lateral distances of 0.42–1.08 m and 0.49–1.03 m, respectively. The vehicle’s oscillation amplitude increased compared to the 90 km/h speed, and there was a slight tendency to drift toward the left lane line after entering the curve, but Vehicle B’s lateral control system was able to suppress the drift and keep the vehicle within the lane.

Based on the analysis shown in Figure 8 and Figure 9, it can be concluded that when entering a curve at high speed, both vehicles showed a tendency to drift out of the lane. This is because, as the curve’s curvature increases, the centrifugal force on the vehicle gradually increases, causing the vehicle to drift toward the left lane line. The lateral control system can suppress the tendency to drift out of the lane, reducing the degree of drift, but it cannot completely eliminate the drift tendency [24]. At speeds of 90 km/h and 120 km/h, as the road curvature increases, the vehicle is more likely to drift out of the lane, and even when it does not drift out of the lane, the oscillation amplitude is large [25]. This trend was observed in both test vehicles, but Vehicle B’s control capability was relatively better. From the same road curvature perspective, as the speed increases, Vehicle A’s oscillation amplitude and drift amount increase, while Vehicle B is able to maintain its position in the curve with a smaller oscillation amplitude. Overall, Vehicle B’s driving automation capability covers more curve scenarios.

Further analysis of the lane-departure test results allows for the calculation of the lateral distance and time at which the lane-departure warning was issued, as shown in Table 7. Vehicle A issued a warning at a lateral distance of 0.18–0.21 m, with a corresponding departure time of only 0.48–0.79 s. Vehicle B issued a warning at a lateral distance of 0.36 m and a departure time of 0.47 s in scenario C3. This indicates that the takeover prompt strategies of both vehicles in lateral control or lane-departure scenarios do not provide sufficient time for the driver to safely take over the vehicle based on the prompt, requiring the driver to continuously monitor changes in road conditions.

### 3.2. Analysis of Longitudinal Control Capability Test Results

**Figure 10:**
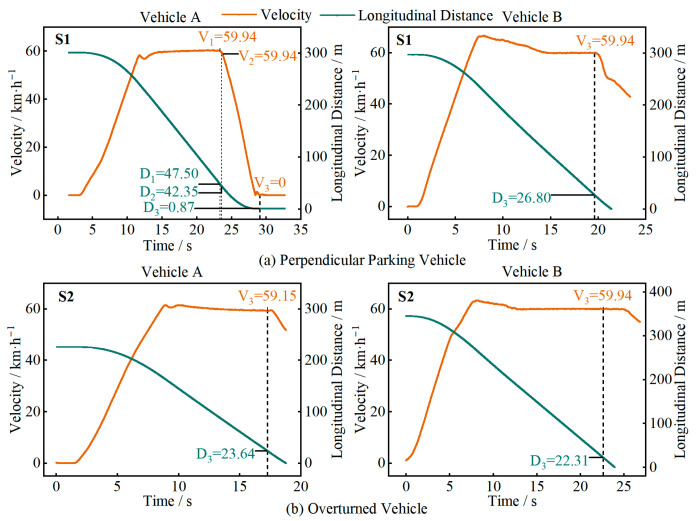
Test results for different vehicle orientations.

**Figure 11:**
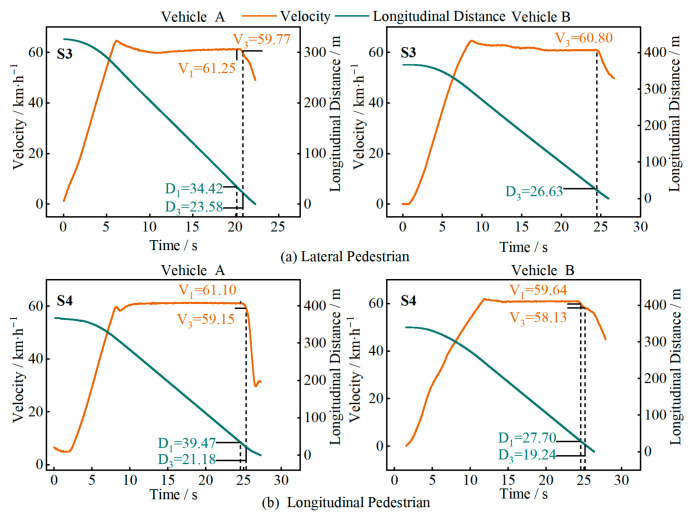
Test results for different pedestrian orientations.

**Figure 12:**
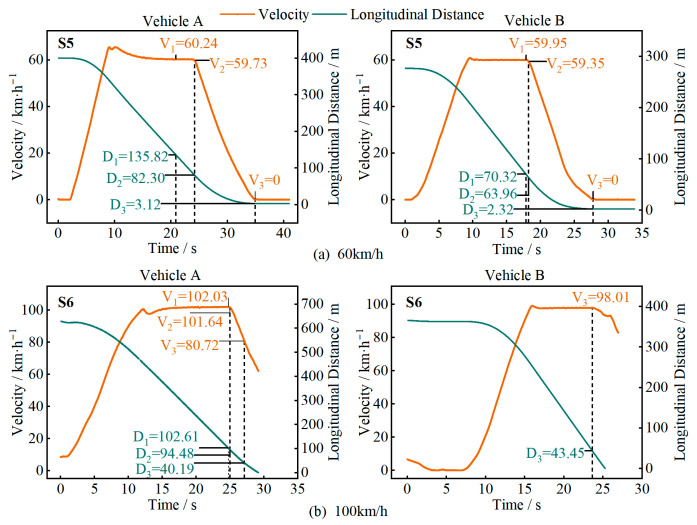
Test results for different speeds involving the vehicle and pedestrian.

**Figure 13:**
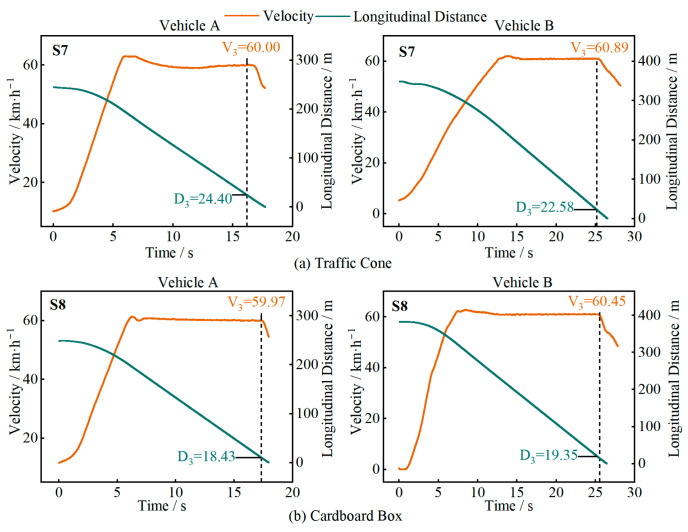
Test results for different objects.

In Figure 10, Figure 11, Figure 12 and Figure 13, $D_{1}$ is the longitudinal distance between the main vehicle and the target when the system issued a warning, and $V_{1}$ is the corresponding main vehicle speed; $D_{2}$ and $V_{2}$ are the longitudinal distance and speed when the vehicle started to decelerate; $D_{3}$ and $V_{3}$ are the longitudinal distance and speed when the vehicle came to a stop or the test driver took over.

In the stationary-vehicle test scenario (S1), as shown in Figure 10a, Vehicle A’s longitudinal control function maintained a speed of 60 km/h and was able to recognize, decelerate, and stop, with both audio and visual warnings. Vehicle B was unable to recognize the stationary vehicle and did not issue any warnings during the test. Figure 10b shows the test scenario with an overturned vehicle (S2), where both vehicles were unable to recognize the overturned vehicle and did not issue any warnings. The possible reason for the difference in recognition between stationary and overturned vehicles is that the vehicle’s perception system has not been trained to recognize overturned vehicles. During the testing, when the driving automation system was unable to effectively avoid a collision, the driver took over the vehicle in time to prevent a collision.

In the stationary pedestrian-crossing test scenario (S3), as shown in Figure 11a, Vehicle A was able to recognize the pedestrian and issued both audio and visual warnings, but did not decelerate. Vehicle B was unable to recognize the pedestrian and did not issue any warnings. Figure 11b shows the test scenario with a stationary pedestrian along the road (S4), where both vehicles performed similarly, recognizing the pedestrian and issuing both audio and visual warnings, but did not decelerate, and the test driver took over.

Figure 12 shows the test results for the combined stationary-vehicle and pedestrian scenario (S5 and S6), simulating a situation where a vehicle breaks down and passengers are at the rear of the vehicle. At a speed of 60 km/h, both Vehicle A and Vehicle B were able to come to a stop, with both audio and visual warnings, stopping at distances of 3.12 m and 2.32 m from the target, respectively. At a speed of 100 km/h, Vehicle A was able to recognize the target and decelerate in time, but due to the high speed, it was unable to come to a complete stop and issued a warning. Vehicle B was unable to recognize the target and did not issue any warnings.

Figure 13 shows the test results for two types of irregularly shaped targets (S7 and S8). At a speed of 60 km/h, neither test vehicle was able to recognize these stationary targets. These targets are irregularly shaped, small in size, and low in height, making them prone to misidentification during driving, posing a potential collision risk.

By selecting the distance and speed data corresponding to different coordinate points in the longitudinal control test results and using Equations (2) and (3), the takeover reaction time can be calculated, as shown in Table 8. In scenarios where the target was recognized (S1/S3/S4/S5/S6), Vehicle A’s average warning time was 3.79 s, 0.84 s longer than Vehicle B’s 2.95 s, indicating a significant difference. This result shows that there is a significant difference in the target-recognition capabilities and takeover warning strategies between the two vehicles. Since different drivers have varying response times to system warnings and takeover prompts, the design differences in warning and takeover strategies can directly affect the takeover safety of drivers in human–machine cooperative driving scenarios [26]. To further explore the relationship between warning time, driver takeover reaction time, and safety, a detailed analysis of the warning and takeover strategies of the two vehicles is needed.

## 4. Discussion

**Figure 14:**
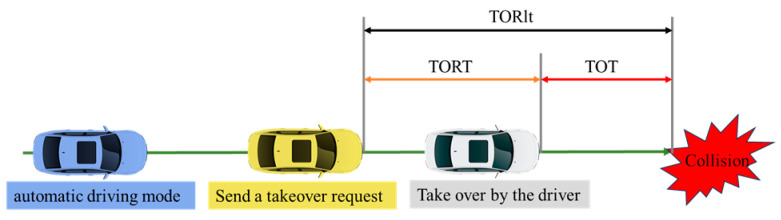
Schematic diagram of different types of takeover times.

Takeover reaction time (TORT) is typically defined as the time from the issuance of a takeover request (TOR) to the driver initiating control. The time from the driver taking over to the collision is referred to as takeover time (TOT), while the total time from the issuance of the TOR to the collision is known as TOR lead time (TORlt). Research indicates that the average TORlt for drivers ranges from 3 to 7 s [27], and the average TORT is approximately 2.72 s [28], which is generally considered the minimum time required for a driver to safely regain control and avoid a potential collision, as illustrated in Figure 14.

Based on the real-vehicle testing in this study, Table 7 shows the lateral distance and departure time when the vehicle issued a TOR. In the three functional-boundary scenarios that caused lane departure, Vehicle A issued a TOR at an average lateral distance of $\bar{d_{f}}$ = 0.2 m, with an average departure time of $\bar{T_{f}}$ = 0.56 s, meaning the TORlt was 0.56 s. Vehicle B issued a TOR at $d_{f}$ = 0.36 m, with a TORlt of 0.47 s. This means that if the drivers rely on the TOR to take over the vehicle, they must recognize the functional boundary and take over within $T_{fmin}\in$ (0.47, 0.56). Clearly, this warning time poses a significant risk, as it is difficult for drivers to safely take over in such a short time in real-world use. This indicates that both vehicles need further optimization in their lateral control takeover prompts. Notably, during testing, whenever the vehicle issued a lane-departure warning, the lane-keeping and departure-prevention functions were immediately deactivated. For Level 3 and above driving automation functions, the relationship between warning design and function deactivation mechanisms must be reconsidered to avoid vehicle loss of control [29].

Based on the longitudinal control test results, Table 8 shows the distribution of TORlt (takeover time lead $T_{1}$), TOT (takeover time $T_{3}$), and TORT (takeover reaction time ∆T). In scenarios where the vehicle came to a stop, Vehicle A’s TORlt was 2.85 s (S1) and 8.12 s (S5), while Vehicle B’s TORlt was 4.22 s (S5), all within the safe takeover time range. In scenarios where the vehicle did not come to a stop, Vehicle A’s TORlt was 2.02 s (S3), 2.33 s (S4), and 3.62 s (S6), with TORTs of 0.60 s (S3), 1.04 s (S4), and 1.83 s (S6). Vehicle B’s TORlt was 1.67 s, with a TORT of 0.48 s (S6 speed was 100 km/h, others were 60 km/h). These TORlt and TORT values exceed the safe takeover time range [27,28]. Drivers using assisted driving functions are prone to passive fatigue and over-reliance on TOR [30], and insufficient warning strategies can lead to serious consequences. Additionally, conditional automated driving allows drivers to engage in non-driving-related tasks (NDRTs), leading to longer TORTs [31]. Therefore, intelligent vehicle accident investigations should focus on the rationality of the system’s warning prompts and warning strategies, such as the product defect issues involved in the recall of Tesla’s Level 2 driving-assistance functions [32,33].

From the perspective of takeover strategy design, existing research has paid little attention to the process after takeover in hazardous scenarios. In the testing of the two vehicles, the average TOT for the nine unrecognized scenario events was 1.40 s, which can be considered the professional driver’s limit takeover time before a collision. Combined with the shortest departure time $T_{fmin}\in$ (0.47 s, 0.56 s), the range of time needed for the driver to safely take over is (1.87 s, 1.96 s) in the lateral control state. Combined with the basic safety TORT of 2.72 s [28], the minimum takeover time for a driver to safely take over (avoiding a collision and having enough time to observe the surroundings) in a longitudinal control state is 4.12 s.

The majority of extant studies on driver takeover time are based on driving simulator experiments, while data from real-world vehicle tests remain relatively limited. Studies conducted using simulators have demonstrated that the duration of takeovers typically ranges from 3 to 8 s [34]. It has been demonstrated that extended takeover periods contribute to enhanced driving safety and facilitate the alleviation of the driver’s mental workload [35]. However, the virtual environments created by driving simulators differ significantly from real-world road conditions in terms of the psychological effects they have on drivers, often leading to deviations when simulator results are applied to actual driving scenarios [36]. Conversely, real-world tests have been shown to offer a more accurate reflection of drivers’ behavioral responses in natural traffic environments. Existing real-world studies have indicated that in most takeover scenarios, a warning time of approximately 7 s is sufficient for drivers to complete the takeover process, while 4 s is generally considered the minimum threshold for a successful takeover [37,38]. The empirical test results obtained in this study are consistent with the aforementioned range.

Although standards such as those from SAE and ISO have not explicitly defined specific takeover times, they emphasize that the system should provide a “sufficient warning time” to ensure a smooth transfer of control to the driver [39]. According to SAE Standard J3016 [40], in the context of Level 3 (conditional automation) systems, the issuance of a takeover request (TOR) to the driver is mandated. The driver must be designated as a “fallback-ready user,” thereby signifying their capacity to “immediately resume” vehicle control. In this study, the participants were young and experienced drivers who demonstrated high levels of attentiveness throughout the duration of the test. Consequently, the condition of these users can be regarded as approximating the “fallback-ready user” as delineated by SAE J3016. Consequently, the lateral and longitudinal takeover times measured in this study should be interpreted as relatively short reference values. In real-world driving contexts, significant individual variations in driver skill and attentiveness mandate that takeover strategies be dynamically adapted based on driver monitoring systems (DMS). This enables a balanced approach to safety and efficiency.

In summary, the findings of this study indicate that L2 automated driving systems should provide drivers with the minimum required takeover time when facing lateral and longitudinal driving risks, which holds positive significance for the development of takeover strategies in automated driving systems. However, this study still has certain limitations. (1) In the lateral control scenarios, due to test site constraints, repeated tests were not conducted on left-turn curves, and the parameter settings for the curved-road scenarios had large gradients, making it difficult to fully analyze the effects of curvature and cruising speed gradients on automated driving functions. (2) In the longitudinal control scenarios, the targets used were based on ISO 19206 standards [41], and the diversity of target types could be further expanded. (3) For safety reasons in real-vehicle testing, scenarios involving driver distraction or fatigue were not included in the takeover tests. Future research can enrich the variety of road types and target objects in real-vehicle tests and introduce physiological monitoring devices such as electroencephalography (EEG) and electrocardiography (ECG) to systematically analyze drivers’ takeover capabilities across different genders, age groups, and physiological states. This will further promote the integration of driver monitoring systems (DMS) with takeover strategies and help establish more adaptive and hierarchical takeover systems. Additionally, the test vehicle used in this study was not equipped with LiDAR, which has inherent advantages in obstacle detection. Factors, such as object shape, material, and size, significantly affect perception performance. Future studies incorporating multi-sensor fusion perception schemes are expected to significantly enhance the system’s environmental perception capabilities in complex scenarios.

Research in other fields, extending beyond the automotive domain, has recently proposed optimization and control approaches that may serve as a source of inspiration for future advancements in takeover strategies. For instance, Tan et al. proposed a dual control framework with Nesterov accelerated gradient descent (DCEE-NAGD) for autonomous airborne source search, which effectively balances exploration and exploitation, thereby enhancing search efficiency and robustness [42]. In a similar vein, Hu et al. developed a two-layer optimal scheduling method for microgrids based on adaptive stochastic model predictive control. In this method, adaptive period partitioning and scenario-based constraints significantly reduced the negative impact of uncertainty on system performance [43]. The extant literature indicates that the integration of advanced predictive control and optimization algorithms into automated driving systems has considerable potential. By leveraging such approaches, future takeover strategies can be rendered more adaptive and resilient, thereby enhancing both driver safety and system reliability in complex driving environments.

## 5. Conclusions

Based on in-depth investigations of typical accidents, it was found that some autonomous driving systems have significant functional limitations in curved-road scenarios and static-obstacle scenarios. To explore the functional boundaries of autonomous driving and takeover safety, this study designed test scenarios and conducted real-vehicle testing to analyze the lateral and longitudinal control issues of vehicles in these scenarios. The conclusions are as follows:

1. In scenarios involving roads with large curvatures, such as urban roads and highway ramps, the lateral control capabilities of some driving automation systems are insufficient, potentially leading to sudden system deactivation and lane departure. Additionally, the test vehicles’ ability to suppress drift in the section of the curve with constant curvature is poor, resulting in large oscillation amplitudes within the curve and posing safety risks;
2. In static irregular obstacle scenarios, some driving automation systems have perception limitations, potentially leading to missed target detection or failure to recognize targets, resulting in a lack of effective warnings and takeover prompts for the driver. This can cause the vehicle to fail to decelerate in time, increasing the risk of collision accidents;
3. For Level 2 combined driving assistance systems and other human–machine co-driving stages, vehicle design should focus on enhancing driver engagement in driving tasks, ensuring the driver is fully involved and ready to take over in an emergency. For Level 3 conditional driving automation functions, the system’s early-warning capabilities should be strengthened to ensure the driver has enough time to take over. Research shows that the takeover request for longitudinal control requires at least 4.12 s for the driver to react in time, while the takeover request for lateral control requires at least 1.87 s.

## Figures and Tables

**Table 1:** Vehicle state before collision.

Time (s)	NOA State	Speed (km/h)	Vehicle State	Remarks
−5	Active	111	/	No warning or takeover prompt
−2.4	Lateral control deactivated	111	/	
−1.75	Fully deactivated	111	Lane departure	
−0.75	/	104	Braking, steering	Driver takeover
0	/	87	Collision	Collision with guardrail

**Table 2:** Vehicle state before collision.

Time (s)	NOA State	Distance to Collision Target (m)	Remarks
−10	Active	250	No warning or takeover
−7		174	
−3.69		62	
−1.49	Deactivated	7	Driver takeover
0		Collision	

**Table 3:** Lateral control capability test scenarios.

Test Content	Test Scenario	Scenario ID
Lateral Control	Main vehicle at 60 km/h entering a 250 m radius right curve	C1
	Main vehicle at 90 km/h entering a 250 m radius right curve	C2
	Main vehicle at 120 km/h entering a 250 m radius right curve	C3
	Main vehicle at 90 km/h entering a 500 m radius right curve	C4
	Main vehicle at 110 km/h entering a 500 m radius right curve	C5
	Main vehicle at 120 km/h entering a 500 m radius right curve	C6

**Table 4:** Longitudinal control capability test scenarios.

Test Content	Test Scene	Scenario ID
Longitudinal control ability	Target: stationary vehicle, main vehicle speed 60 km/h	S1
	Target: overturned vehicle, main vehicle speed 60 km/h	S2
	Target: stationary pedestrian crossing, main vehicle speed 60 km/h	S3
	Target: stationary pedestrian along the road, main vehicle speed 60 km/h	S4
	Target: stationary vehicle and pedestrian, main vehicle speed 60 km/h	S5
	Target: stationary vehicle and pedestrian, main vehicle speed 100 km/h	S6
	Target: traffic cone, main vehicle speed 60 km/h	S7
	Target: cardboard box, main vehicle speed 60 km/h	S8

**Table 5:** Sensor and function configuration comparison.

Item	Vehicle A	Vehicle B
Sensor scheme	12 ultrasonic radars 7 cameras 5 mm-wave radars	12 ultrasonic radars 5 cameras 3 mm-wave radars
lateral control	Lane keeping assist system Lane deviation suppression system Lane offset reminder function	Adaptive curve cruise Lane center auxiliary Intelligent auxiliary lane change Lane departure warning
longitudinal control	Active cruise control system Collision mitigation braking system Front collision warning function	Forward collision warning Forward distance monitoring

**Table 6:** Ideal lateral distance range.

Item	Test Vehicle A	Test Vehicle B
w (m)	1.790	1.896
$d_{p}$ (m)	0.665~1.145	0.612~1.092

**Table 7:** Lane-departure warning distance and time in the lateral control capability test.

Scenario	Test Vehicle	$d_{f}$ (m)	$T_{f}$ (s)
C2	A	0.21	0.79
C3	A	0.19	0.42
	B	0.36	0.47
C6	A	0.18	0.48

**Table 8:** Longitudinal control capability takeover reaction time.

Scenario	Vehicle A		Vehicle B		ΔT (s)	
	$T_{1}$ (s)	$T_{3}$ (s)	$T_{1}$ (s)	$T_{3}$ (s)	Vehicle A	Vehicle B
S1	2.85	—	—	1.61	Stopped	Not recognized
S2	—	1.44	—	1.34	Not recognized	Not recognized
S3	2.02	1.42	—	1.58	0.60	Not recognized
S4	2.33	1.29	1.67	1.19	1.04	0.48
S5	8.12	—	4.22	—	Stopped	Stopped
S6	3.62	1.79	—	1.60	1.83	Not recognized
S7	—	1.46	—	1.33	Not recognized	Not recognized
S8	—	1.11	—	1.15	Not recognized	Not recognized

## Data Availability

This study was conducted using data from the NAIS database. Access to the NAIS database is restricted and requires an application or fee to be paid to the relevant authorities, as it is not an open-source database.
